# To Intervene or Not to Intervene: An Experimental Methodology Measuring Actual Bystander Behaviour

**DOI:** 10.3390/bs15040550

**Published:** 2025-04-18

**Authors:** Danielle Labhardt, Nadine McKillop, Emma Holdsworth, Sarah Brown, Douglas Howat, Christian Jones

**Affiliations:** 1 Faculty of Health and Education, School of Psychology, Manchester Metropolitan University, Brooks Building, Manchester M15 6GX, UK; 2 School of Law and Society, University of the Sunshine Coast, Sippy Downs, QLD 4556, Australia; nmckillo@usc.edu.au (N.M.); sbrown2@usc.edu.au (S.B.); cmjones@usc.edu.au (C.J.); 3 Faculty of Health and Life Sciences, Coventry University, Coventry CV1 5FB, UK; aa7076@coventry.ac.uk (E.H.); hsx063@coventry.ac.uk (D.H.)

**Keywords:** sexual violence, bystander intervention, deception, experimental methodology, funnelling debrief

## Abstract

Bystander intervention and sexual assault research typically rely on self-reported intent to intervene. However, predicted behaviour can be considerably different from actual behaviour. Hypothetical scenarios are often utilised to remove extenuating circumstances, limiting insight into actual behaviour where those circumstances impact intervention. This paper discusses the development and evaluation of an innovative methodology to measure actual bystander behaviour when witnessing signs of an impending sexual assault. With careful attention paid to ethical considerations and participant safety, 13 participants were directly deceived about the true aim of the research. Utilising observational data and a funnelling debrief, the findings demonstrated varied reactions to sexual assault cues, from not noticing and therefore not intervening, to noticing and (in)directly intervening. Participants’ responses indicated they remained unaware of the deception until it was formally revealed, suggesting the methodology effectively realised the study’s aims. The funnelling debrief mitigated the adverse effects of the deception, with some participants reporting more confidence and motivation to intervene in the future. Further development of this methodology could create more interactive bystander intervention programmes that teach people to identify signs of a sexual assault, develop skills to safely intervene, and raise awareness about sexual violence.

## 1. Introduction

Initially, bystander research focused on emergencies (e.g., smoke-filled room; Latané & Darley, 1968) and non-emergency situations (e.g., stranded motorist; Hurley & Allen, 1974). In recent years, researchers have focused on sexual assault (see Labhardt et al., 2017; Mainwaring et al., 2023). Typically, this research has relied on self-report data to assess ‘intent to intervene’ using vignettes (e.g., Banyard et al., 2005; Nicksa, 2014; Mainwaring et al., 2023).

Vignettes and self-report measures elucidate how individual and contextual factors impact intent to intervene (e.g., Bennett et al., 2017, 2014; Hoxmeier et al., 2017; Kania & Cale, 2018) without exposing participants to an actual sexual assault. However, this has limitations. First, participants can only identify behavioural intentions; predicted behaviour can be considerably different from actual behaviour (McMahon et al., 2014). Second, vignettes remove extenuating circumstances so the variables of concern can be experimentally controlled. Regarding ecological validity, this limits insight into actual behaviour where contexts vary, and there is a complex interplay between several factors (Hughes, 1998). Third, participants are often aware of the study aim, potentially increasing socially desirable responses (Grimm, 2010). Experimental deceptive research measuring actual behaviour might therefore be more helpful.

Two studies using an experimental deceptive design in the 1980s were identified (Harari et al., 1985; Shotland & Stebbins, 1980). Shotland and Stebbins (1980) deceived participants of the true study aim. They used audio and minimal visual cues to create a sexual assault scenario, e.g., a woman being dragged into a room and shouting, “Help, rape”. As the seriousness and clarity of the situation increased, intervention rates increased, with about 30% of bystanders intervening in the most serious/clear scenarios. Harari et al. (1985) used actors, where a man jumped out of the bushes at night and dragged an unsuspecting woman away while she was walking alone, who then shouted, “Help, rape”. Male bystander reactions were examined. They were only informed of the study once they tried to intervene or at the end of the study, followed by a short debrief. Approximately 80% of men intervened, with the majority directly intervening (approaching the perpetrator and victim).

Shotland and Stebbins (1980) and Harari et al. (1985) examined actual intervening behaviour, but there are some limitations. Sexual assaults are more frequent between people who know each other (e.g., GarcÍa-Moreno et al., 2005; Karjane et al., 2005); yet, in both studies, the victim and perpetrator were portrayed as strangers (Harari et al., 1985; Shotland & Stebbins, 1980). Additionally, the risk of someone jumping out of the bushes is minimal; approximately 69% of sexual assaults in Australia occur in surroundings familiar to either the victim and/or the perpetrator (Australian Bureau of Statistics, 2024), which is consonant with findings in the United Kingdom (Stripe, 2021) and the United States (RAINN, 2018).

Thus, the current study’s aim was to design and evaluate a methodology, with high ethical standards, that assessed actual behaviour in a more typical sexual assault scenario. University students were the focus as they are at an increased risk of sexual victimisation (Ministry of Justice, 2013; Office for Students, 2022; RAINN, 2018; Revolt, 2018).

### 1.1. Design of the Innovative Methodology

The following considerations were made in the development of this methodology in order to test actual bystander behaviour: (1) create a naturalistic environment where sexual assaults are most likely to occur, and (2) encourage authentic responses and behaviour using deceptive methods while maintaining high ethical standards.

#### 1.1.1. Naturalistic Environment

The environment should be as simple and realistic as possible and reflect what naturally happens in sexual assault situations. Only then can actual bystander behaviour naturally evolve. The risk of sexual assault is highest in the 18 to 24 age group when they are attending a party (Australian Bureau of Statistics, 2024; Australian Human Rights Commission, 2017), which is consonant with findings in the United Kingdom (Ministry of Justice, 2013; Universities UK, 2016) and the United States (Sinozich & Langton, 2014); therefore, a party environment was created in the study.

##### Development of the Immersive Studio Methodology

To create the environment, a 7m-by-7m creative space containing six projectors was used, called the Immerse Studio. This environment is safe and controlled (Riva et al., 2015) with a one-way glass observation room. A party environment was created based on interviews conducted with university students (Labhardt, 2019) and discussions with Applied Theatre Performance (ATP: a university acting group) actors about what they have seen at clubs and parties. A DJ wall, using Spotify, was projected on the left wall of the studio. Music was recommended by the ATP actors. A typical university party includes drinking games. As alcohol was not included, an alternative game was required to immerse participants. An XBOX Kinect Motion Monitor was used with the centre wall of the room. Participants selected from several images (e.g., beach scene) on a laptop, connected to the Kinect. The Kinect allowed participants to superimpose themselves within the image, projected on the wall, and take screenshots of themselves. The right wall was a selfie wall. Participants could take photos alone or with others using an iPad. Photos were uploaded to a private, closed website and projected onto the wall, where they continuously circulated. These interactive features facilitated a realistic party environment, encouraging participants to enjoy themselves and interact with each other and the technology. Snacks, water, and an alcohol-free party punch were also provided, contributing to the party environment.

#### 1.1.2. Deception

Using direct deception, participants were told the focus of the study was to understand the utility of the Immerse Studio for hosting social gatherings, such as on-campus parties. However, unbeknownst to the participants, actors were deliberately placed to portray sexual assault cues. The debate around using direct deception has been ongoing (for a detailed discussion, see Boynton et al., 2013). Direct deception is when participants are deliberately misinformed about the study, including but not limited to study descriptions, instructions, or the use of actors.

Those against deceptive research argue that deception negates participants’ autonomy as they are not fully informed about the nature of the study and thus cannot fully consent, and that deceptive research harms participants’ emotional state and self-esteem (Benham, 2008; Bortolotti & Mameli, 2006; Boynton et al., 2013). However, others argue deceptive research is possible if it is used to answer questions that non-deceptive research cannot answer and the findings make a significant contribution to scientific, educational, and practical knowledge (American Psychological Association, 2002; Benham, 2008; Boynton et al., 2013; Jamison et al., 2008). Furthermore, deception should be revealed as soon as possible, using a comprehensive and thoughtful debrief (Benham, 2008; Boynton et al., 2013). Researchers must reiterate that participants can withdraw at any point, including after revealing the deception, allowing them to exercise their autonomy (Boynton et al., 2013). If these conditions are met, deceptive research can be conducted to allow for true reactions to be measured (Tai, 2012).

Several factors were considered to maintain participant well-being throughout participation. The first was the depiction of the sexual assault in the party environment. It is acknowledged that it is likely that participants may be survivors or know someone who is a sexual assault survivor. As such, to minimise harm to participants and create a realistic scenario, the sexual assault depicted by the actors only included signs leading up to a sexual assault, similar to what is typically seen on TV, movies, or a night out (Labhardt, 2019).

The second consideration was participants’ mental well-being. University Student Wellbeing was notified of the project and was on-call when data were collected. A registered psychologist and social worker were present on the day for any participants experiencing adverse reactions following participation. To ensure the actors’ safety, one actor, apart from the victim and perpetrator, would leave the room to signal a problem, and campus security was on call for potential risks, including attacks on the actors. Participants met the actors post-participation, allowing participants to understand that the actors were acting and offering an opportunity for discussion of the experience, further reducing possible adverse reactions.

The third consideration was that participants received a comprehensive debrief. The funnelling debrief method is recommended for direct deception research (Boynton et al., 2013). This method acts as a conversation between the interviewer and participant, revealing the deception and true aim of the research naturally, mitigating negative effects, and maintaining methodological integrity.

Past bystander research (Schwartz & Gottlieb, 1980) has demonstrated that deceptive research is beneficial. Although there may be a short-term reduction in intervention, the likelihood of intervening increases after approximately six months. Furthermore, including a detailed funnelling debrief has been shown to have a strong positive effect for participants (Boynton et al., 2013), suggesting a positive effect on intervening behaviour in the long term.

##### Actors

Invisible theatre (Boal, 1985, 1992) was used to facilitate the signs leading up to a sexual assault. Actors play everyday people using improvisation, ensuring viewers are unaware that the drama is scripted. Actors work from a ‘toolbox’ of required behaviours that need to be performed. These behaviours are presented organically to ensure naturalistic interactions.

Four actors (two men and two women) from the ATP team, aged 18–25 (similar to the participants), improvised the signs leading up to a sexual assault. One female actor, posing as a project volunteer, introduced and demonstrated the technology to participants and assisted throughout. The other three ATP actors posed as participants, arriving at the same time as the actual participants, ensuring participants saw them as fellow participants. However, in addition to their roles as participants, one male ATP actor encouraged people to interact if needed, while the last two played the roles of the female victim and the male perpetrator. Worldwide statistics demonstrate women are most at risk of experiencing sexual violence by men (World Health Organization, 2021); therefore, for this study, the male perpetrator and female victim narrative was utilised.

The victim and perpetrator characters were developed from interviews conducted with university students (Labhardt, 2019). The victim played a friendly, shy, and polite character who thought she might have something in common with the perpetrator. The perpetrator played a confident, arrogant character used to getting his way.

##### Signs Leading up to a Sexual Assault

The perpetrator and victim acted out behaviours leading up to a sexual assault, developed specifically for this project from interviews with university students (Labhardt, 2019). Table 1 provides an overview of the behaviours. In line with the invisible theatre approach, these behaviours were presented organically, increasing in severity as the situation developed.

**Table 1:** Basic behaviours signalling signs leading up to a sexual assault, as depicted by the victim and perpetrator.

**Figure 1:**
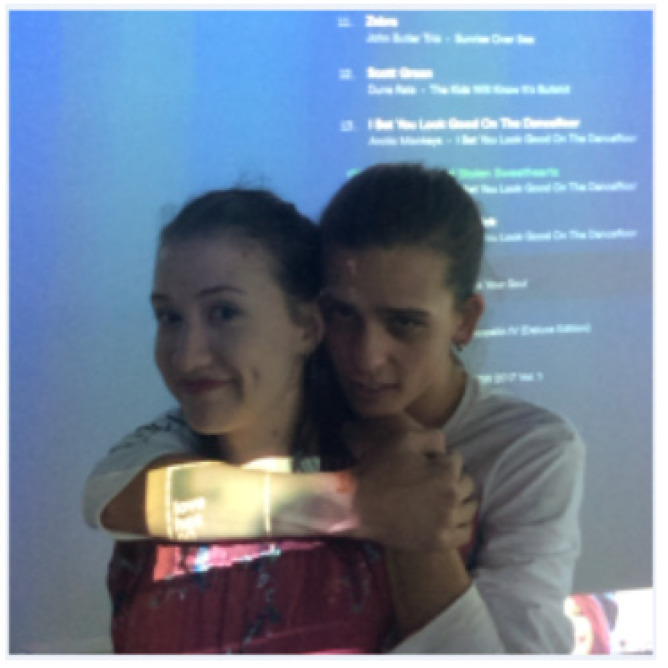
An example of a ‘creepy’ or inappropriate photo that was taken at the ‘party’.

The Arrival. Victim comes in with a friend (actor). Perpetrator enters last. Perpetrator and victim are introduced by a mutual friend (actor). General discussion (perpetrator checked control room). Perpetrator and victim take a selfie. Perpetrator gets victim a drink. Second drink or forcing to down the first drink. Victim moves away, perpetrator follows and encroaches on personal space. Perpetrator takes inappropriate photo with victim and uploads it to the selfie wall. (See Figure 1 for image). Victim tries to leave, she is visibly upset, the perpetrator wants to “hug it out”. Victim leaves, perpetrator follows to “see if she’s ok”.

## 2. Method

Ethical approval was obtained, and the party was ‘held’ twice. There were three research questions:

1. How do participants respond to signs leading up to a sexual assault in a party environment?
2. How successful is the methodology in creating a believable party environment to test bystander behaviour via deception?
3. How do participants perceive the use of deception?

Research questions 1 and 2 were addressed via observations and interviews, while research question 3 was addressed through post-deception/debrief interviews.

### 2.1. Participants

In accordance with Terry et al. (2017) and Hennink and Kaiser (2022), 13 students (*M_age_* = 20.92, *SD* = 2.02, *Range* = 18–24) from an Australian University were recruited. No participant withdrew during or after participation. Participants included four males and nine females. The majority (92.3%) identified as White (*n* = 12), with one (7.7%) Asian. All participants were undergraduate students; 23.1% studied psychology (*n* = 3), and 30.8% studied a combined degree of criminology/psychology (*n* = 2) or serious games (*n* = 2). The remainder were across criminology (*n* = 1), psychology and counselling (*n* = 1), engineering (*n* = 1), IT (*n* = 1), personal training (*n* = 1), and teaching (*n* = 1).

Participants were allocated to one of two weekend sessions (09:30 or 12:30). While not planned and purely coincidental, approximately half of the participants in each session knew each other. The first session consisted of four participants: two were in a relationship and knew two of the actors (either as an acquaintance or had seen them in a recent theatre production), but did not suspect that they were there in an acting capacity. The second session consisted of nine participants: two were in a relationship and knew one other person in the session, and two were siblings.

#### 2.1.1. Funnelling Debrief Semi-Structured Interview Schedule

There were six interviewers simultaneously conducting the funnelling debrief to minimise the wait time between the ‘party’ (part 1) and the group debrief (part 3). The funnelling debrief utilised a semi-structured interview style. The funnelling debrief started out with broad questions on what participants thought of the overall experiment, what they found positive about the experience, and what they found negative about the experience. Based on these questions, the interview could go one of four ways depending on participant responses: (1) Participant raised concerns, unprompted, about observing sexual assault cues. The interviewer asked the participant to elaborate on what they found concerning, if they acted on their concerns, and why; (2) if the participant did not mention the sexual assault signs, the interviewer prompted the participant by telling the participant that concerns were raised about some uncomfortable/awkward behaviour in the room. The interviewer asked if the participant noticed the behaviour. If this sparked the memory and participants then reported noticing the uncomfortable/awkward behaviour, they were asked to elaborate, if they acted on their concerns, and why; (3) participant was prompted (as described in the second option, preceding this one) but unlike the second way, these participants reported that they were not aware or notice any uncomfortable/awkward behaviour; and (4) only for participants who directly intervened or sought out the lead author as a method of intervening, the interviewer asked them to elaborate on what they noticed or were concerned about, what made them feel that way, and what influenced them to act on their concerns. Following this, the interviewers revealed the deception and the true research aim. Interviewers guided participants to reflect on what this meant and how they felt about it, and participants were reassured that their responses were completely normal, the deception was necessary, and that their contribution was invaluable.

#### 2.1.2. Post-Deception Consent

Once the deception was revealed, participants were provided with a post-deception consent form, allowing them to decide whether their data would be included in the research. Participants were informed that if they withdrew, their individualised responses (i.e., questionnaire and interview) would be deleted. However, as this was a social experiment involving a group of participants, data resulting from the video could not be withdrawn, but any reference to what they did in the Immerse Studio would not be used.

#### 2.1.3. Live Video Feed

**Figure 2:**
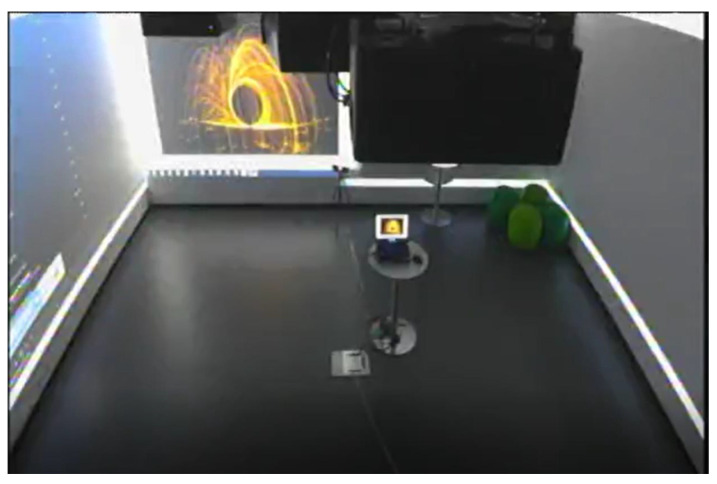
Camera in the centre of the back wall.

**Figure 3:**
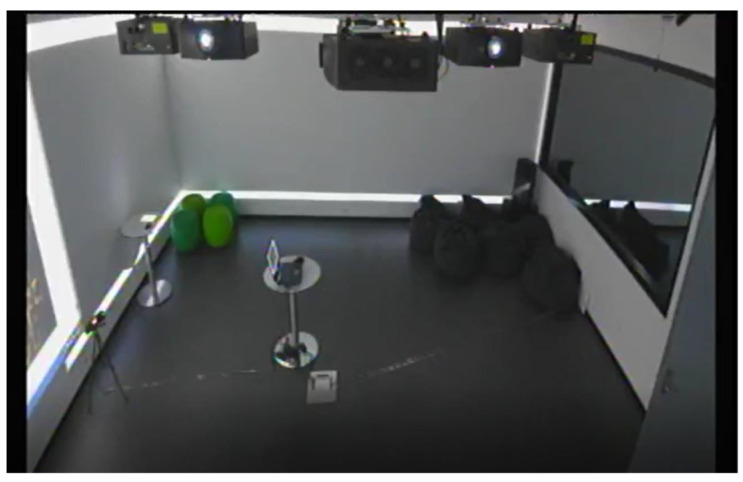
Camera on the left side, closest to the door.

All activity in the Immerse Studio was observed via B-Line (2017), a live multi-angle video feed, in the Engage Lab across from the Immerse Studio. There were two cameras in the Immerse Studio. Camera one (see Figure 2) captured most of the three projector walls. Camera two (see Figure 3) captured the Kinect Motion Monitor, the selfie wall, and the back wall. All activity was recorded so that data could be analysed and coded later.

#### 2.1.4. Coding Scheme for Video Recording

**Figure 4:**
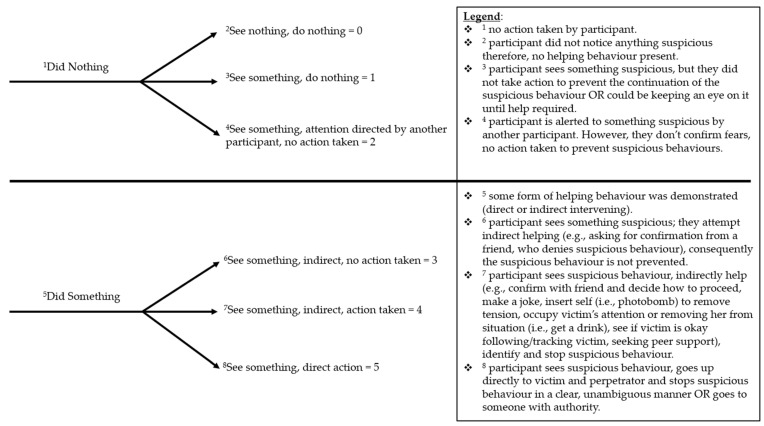
Coding and scoring scheme for intervening behaviour.

A coding scheme was developed to record participants’ behaviour during the session (see Figure 4). Codes were divided between “did nothing” (i.e., no intervening behaviour) and “did something”, where some form of intervening behaviour was identified. From there, codes were created to explain the type of intervention (e.g., indirect or direct intervention). Coding was completed separately by the lead author and the third author. The authors met and discussed the coding; any discrepancies were discussed before agreeing on a final code.

### 2.2. Procedure

Utilising opportunity sampling techniques, participants were recruited using BlackBoard (the university’s online student communication tool), University Facebook groups, posters, and face-to-face recruitment at Orientation Week. Participants were deceived from the onset, believing the study was about how effective the Immerse Studio was at hosting social gatherings such as parties. Participants expressed interest via email, whereupon additional information was emailed to them, and they were offered a time slot, 09:30 or 12:30, on a Saturday. Each session had three parts: (1) Immerse Studio, (2) questionnaire and interview, and (3) group debrief.

Participants and three of the ATP actors (detailed in Section Actors above) arrived together for their allocated time slot. The lead author introduced participants to the volunteer (the first ATP actor), who introduced the Immerse Studio and the activities in the room. The lead author then left the Immerse Studio. The “party” lasted approximately 20 min, with the ATP actors performing the scripted scenario. All activity was monitored via video feeds to ensure the safety of participants and the ATP actors. It also allowed interviewers to focus on the observed behaviours of participant(s) they would later interview. The session ended when the sexual assault signs escalated to the point where the ATP actor portraying the victim left the room, and the ATP actor portraying the perpetrator followed her out under the guise of making sure she was okay. Participants were given one minute to react. Following this, the lead author entered the room to move the study to the next stage.

To complete the questionnaire and interview (part two), participants were directed to separate rooms to remove the possibility of discussing what happened. Participants completed an anonymous demographics questionnaire on an iPad. Next, participants were interviewed using the funnelling debrief method. After the deception and true aim of the project were revealed, participants were provided with a post-debrief informed consent form and a formal debrief form with available support in case they or someone they know required it. Additionally, participants were informed that a registered psychologist and a social worker were available if they wanted immediate support; two participants utilised this support. Both participants reported feeling better after their discussions. Participants were compensated AUD 20 for their time. The questionnaire and interview lasted approximately 10–20 min.

Once the interviews were completed, participants moved to part three, the group debrief. They were taken back into the Immerse Studio to meet the actors. Participants as a group partook in a conversation with the ATP and research team, lasting approximately 10 min. Participants reflected on the overall experience, what they thought about the acting, whether the experimental design was believable, and whether the overall experience felt naturalistic and realistic. Each session lasted approximately one hour. Lastly, participants were emailed after three days to thank them for participating and do a wellness check. Two participants responded with positive feedback. No negative responses were received.

### 2.3. Data Analysis

All interviews were transcribed verbatim and analysed using inductive reflexive thematic analysis, where the analysis was data-driven (Braun & Clarke, 2021). This allowed the research team to work collaboratively and reflexively to identify patterns and relationships within the data, ensuring a more nuanced representation of the data. The six phases, as detailed by Braun and Clarke (2021), were adhered to when analysing the data. Transcripts were read and re-read until familiar with the data. Initial codes were created by the lead author and reviewed by the research team. Codes were assembled and analysed into themes. Themes were reviewed to ensure they accurately reflected data and were presented logically to best present the findings. All authors reviewed, refined, and agreed upon themes and extracts. All extracts were presented with a pseudonym, gender, and session time (i.e., 09:30 or 12:30).

## 3. Results

Research question 1 was answered via observational data and thematic analysis from post-intervention interviews.

### 3.1. Observed Intervening Behaviour

Four of the six possible codes were allocated when coding the videos (see Table 2). Five participants did not witness any sexual assault cues and consequently did not intervene; they were given a code of 0. Five participants saw something but did not intervene and were allocated a code of 1. One participant was given a code of 2 as another participant directed her attention towards the sexual assault cue, but she did not react. Lastly, two female participants in the 12:30 session were given a code of 5 as they noticed the behaviour and took direct action.

### 3.2. Reactions Towards the Sexual Assault Cues Varied

Participants’ reactions towards the sexual assault cues while still under deception varied and were dependent on whether the participants observed the (intended) behavioural cues in the Immerse Studio.

#### 3.2.1. When Prompted: Lack of Awareness and Ambiguity Resulted in Inaction and Feelings of Guilt

Nine participants were prompted during the interviews regarding the sexual assault cues present at the party. Responses for why the sexual assault cues were missed or not mentioned varied from not noticing due to distractions to the ambiguity of the situation. Some participants, primarily from the 12:30 session, reported not spotting any signs related to a potential sexual assault (code 0 in Figure 4).

“*Yeah, so because it was a bigger group, you’re not going to be looking at everything. […] Unless you’re standing back in a corner watching everyone. You’re not really going to pick up on anything unless there was something that would majorly stand out*”.

Jade, Female, 12:30

Jade states that unless a person is looking for something that is not right, there is less likelihood of spotting the signs leading up to a sexual assault. Perhaps the larger group size in session two created a natural distraction that would be present at a real party.

Conversely, some participants did spot something; however, due to the ambiguous nature of the events, these participants perceived the victim and perpetrator to be in a relationship (Code 1 in Figure 4).

“*Yeah, because it’s a situation where you are put in an uncomfortable situation where you don’t know if they are partners and they’re just having a little tiff or like you don’t know*”.

Jade, Female, 12:30

Jade was unsure whether there was a relationship between the victim and perpetrator, which caused hesitation in intervening due to the fear of misinterpretation.

For some participants, the lack of perceived distress from the victim stopped them from intervening.

“*I noticed that he was being very physical with [victim]. Obviously, they hadn’t met before, but she didn’t seem to be too bothered by it. […] it didn’t seem like she was not enjoying his company. So, I was like its fine*”.

Zachary, Male, 09:30

Zachary noticed the perpetrator’s physical behaviours towards the victim. However, he did not perceive the victim’s body language as distressed or wanting out of the situation. The ambiguous body language could cause misinterpretation and result in no intervention.

Most of the participants who were prompted, however, expressed feelings of guilt and remorse for not acting.

“*I feel upset actually. A little upset. To a point I noticed them, but I didn’t notice what was going on*”.

Aidan, Male, 12:30

“*I feel kind of weird now. Because I was aware that something was going on, but […] I was fairly sure that nothing terrible was going on. […] I couldn’t see many signs that she was distressed at the time as well. Ya. I feel pretty bad now*”.

Sophia, Female, 09:30

#### 3.2.2. When Unprompted: (In)direct Action Taken When Witnessing Sexual Assault Cues

Unprompted during interviews, four individuals reported seeing suspicious signs. However, the methods of action taken varied from indirect action that did not result in suspicious behaviour being prevented, to direct intervention taken. Two individuals who used indirect action with no further action taken (code 3 in Figure 4) highlighted the ambiguity of the situation.

“*Well, there was one incident in there where a girl looked really uncomfortable. […] I didn’t know if they knew each other (victim and perpetrator) […] She went up to another guy and said I’m leaving now, and he just looked concerned as well*”.

Georgia, Female, 12:30

Georgia noticed the negative body language depicted by the victim, suggesting something was not right. However, something prevented Georgia from intervening. Perhaps, a fear of misinterpreting the situation.

Alternatively, some felt that not enough time was allocated for bystanders to react to the situation once the victim and perpetrator left.

“*The boy threw an arm over the girl and had a selfie like that (“creepy picture”), and the girl was looking slightly awkward when the photo came up on the wall. […] the girl rushed out and the boy followed after her […] us girls (Mikayla) were saying we had girl-ish intuition that something’s not quite right […] another 5 min and […] we might have gone out*”.

Christine, Female, 09:30

Christine clearly noticed the sexual assault, referring to ‘girl-ish intuition’ as an explanation for how she knew something was not right. However, Christine mentions having five more minutes with no return of the victim and perpetrator would have prompted her to check on them. While longer reaction times could be beneficial, research suggests that if the situation is perceived as serious, reaction times are around 30 seconds (Hortensius et al., 2018).

Lastly, two female individuals with no prior relationship used direct intervention and intervened using multiple methods (code 5 in Figure 4). Both participants mentioned it was clear something was wrong.

“*He (perpetrator) was acting a bit strange and me and my mate (Gabrielle) that I just met there. […] were keeping an eye on him and his interaction with this other girl. Cause I thought it was a bit off and he started to get a bit um, weird and fishy looking*”.

Summer, Female, 12:30

Summer said that something about how the perpetrator interacted with the victim was suspicious. She referred to it as ‘weird and fishy looking.’ One of the initial interventions was indirect:

“*When I first saw him, he handed the girl a cup of cordial and I joked and said haha it could have been roofied and he gave me a really bad look and I was like it’s a joke, it’s a joke, it’s a joke. […] when they stormed off out of the room, that’s when we sort of thought that we should do something*”.

Summer, Female, 12:30

The perpetrator’s reaction to the ‘joke’ she made and how he followed the victim out at the end escalated the severity. For Summer, the signs were obvious, and intervention was needed.

Summer and Gabrielle monitored the situation throughout the party. Both used indirect intervention methods, such as photo bombing a selfie between the victim and perpetrator to decrease tension and using non-verbal cues to check that the victim was okay.

“*This guy he was putting his arm around her, and she would sort of push it away kind of thing. And I could see that she kept walking away and he kept following. So, yeah, I just thought it was a bit intense and I sort of looked at her and gave her a thumbs up, sort of are you okay and she looked a bit uneasy. He ducked off for a second, so I went over and talked to her and tried to hang out with her for a little bit to steer him off. Didn’t work too well. He was pretty persistent*”.

Gabrielle, Female, 12:30

The victim’s uneasy body language prompted more face-to-face interaction between the victim and the bystanders.

When directly intervening, the two participants followed the victim and perpetrator out together.

“*Very shortly after that, she ran out of the room, he ran after her very angry and I didn’t want to leave on my own. So, I got Gabrielle and bolted out of there to see what was going on*”.

Summer, Female, 12:30

Summer describes the perpetrator as angry. Knowing something was wrong, the bystanders followed the victim out together, leaving together as safety in numbers.

Not only was the severity of the situation a trigger point to intervene, but self-perceived age differences between themselves and the rest motivated these two bystanders to act.

“*I think also another thing that might come into it is potentially age. I think, I had a few years off uni, I’m in my third year now and I’m a lot older, maybe a few years older than a lot of other people. […] Like if these kids were adults, I don’t know if I would have intervened*”.

Gabrielle, Female, 12:30

While there were no significant age differences between participants, Gabrielle perceived there to be one. Perhaps this perception made her feel more responsible for the well-being of the victim, prompting her to find support and directly intervene to prevent a possible sexual assault.

### 3.3. Use of Immerse Studio to Create a Believable Party Environment

While participants were still under deception, the study investigated how believable the party environment was as a scenario for testing actual behaviour (research question 2). Participant responses under this theme demonstrated that the deception was successfully maintained throughout the study. Participants were solely focused on reporting their thoughts around the party environment, with no suspicions raised that something ulterior was going on. These responses also provided practical avenues for improving the test environment for future deception studies.

#### 3.3.1. Engaging Party Environment

Participants had a positive impression of the Immerse Studio and the party environment. Participants found the environment conducive to facilitating social interaction.

“*It was pretty fun. […] it’s nice to get to know some people because I don’t exactly go out that much and I don’t meet a lot of new people […] But it was nice meeting a couple new people*”.

Christine, Female, 09:30

Other participants focused more on the food and technology.

“*Yeah, the iPad, you had food there which was more of an opening environment. […] The people were my age group, […] you had the technology. In our generation we are all equipped to that technology*”.

Jade, Female, 12:30

Participants’ authentic responses, with the focus on the familiarity of the environment, the welcoming space, being surrounded by similar-aged people, the food, and the technology (e.g., the iPad was part of the selfie wall), suggest that the deception was effectively maintained. Furthermore, the space itself is also effective at maintaining deception, putting people at ease, and making the situation feel more natural.

#### 3.3.2. Utilising the Space Better to Enhance the Party Environment

Participants provided suggestions to enhance the party environment, which may be helpful for future deception studies. For example, Zachary felt that the technology was not used to its full potential.

“*Maybe not dedicating an entire wall to it (DJ Wall). Maybe have some cool music visualisers instead because now a third of the room is a playlist*”.

Zachary, Male, 09:30

Some participants from the first session, comprising four participants plus the actors, felt the small number of people present was a limitation.

“*I think slightly the amount of people that we had today had an effect. Because there were too little people*”.

Christine, Female, 09:30

While the deception was effectively maintained, the small group size may have limited conversation and negatively impacted the party atmosphere.

Lastly, most participants felt that the party was too short.

“*It was a bit short I felt. I didn’t get to talk to everyone*”.

Oscar, Male, 12:30

Oscar felt that 20 min was not long enough to get to know everyone. These responses demonstrate that the deception was maintained as participants were solely focused on making suggestions to enhance the believability of the party environment.

### 3.4. Participants’ Satisfaction and a Successful Methodology

This theme relates to participants’ reactions to learning about the deception, following full disclosure (research question 3). Findings indicated that the study’s unintended outcome was the opportunity to provide a positive, experiential learning environment for raising awareness and enhancing confidence in bystander intervention.

#### 3.4.1. Undetected Deception

Participants did not detect the deception until it was explicitly revealed.

“*You guys did an amazing job because it was very discreet, […] I didn’t pick up on it*”.

Jade, Female, 12:30

The deception appears to have been effective, allowing the research team to measure actual bystander behaviour regarding signs leading up to a sexual assault.

#### 3.4.2. No Reported Distress from Learning About the Deception

All participants appeared to be okay after the funnelling debrief and the true study aim disclosed. A small sample reassured the interviewer that they were fine with everything.

“*Yeah, I am fine. It was interesting*”.

Zachary, Male, 09:30

“*No, thank you for the opportunity, it was fun*”.

Summer, Female, 12:30

Participants found the experience an interesting learning opportunity.

“*It’s a study worth doing. Congratulations*”.

Zachary, Male, 09:30

“*No, I think it was a really interesting study. Because I’m doing psychology and criminology, so I think it’s definitely. I don’t know, I thought it was really interesting. I think you guys did a good job*”.

Alannah, Female, 12:30

Participants stated that they were glad that this research was being conducted. Two participants emailed the research team after their participation. One provided a reflection of her experience and what it meant for her.

“*I think this is a great and very important study and I hope your paper gets published and gets the attention it deserves so more studies like this can be done. Ultimately, I came for the $20 voucher, but I can say I’ve left with more. I feel more confident and more motivated to do something. If I see someone looking uncomfortable because of another person*”.

Mikayla, Female, 09:30

Mikayla reflected on how her perceived confidence and motivation to do something had increased due to this research. Arguably, this type of research could be beneficial in developing knowledge and confidence to increase bystander intervention when witnessing a sexual assault.

The second participant emailed following participation about her perceptions of the value and significance of this type of research.

“*Your study was amazing and gave me heaps of food for thought—if you ever consider doing another session please do tell me, I’ll try and get you more participants. I really do think that it was a great experience and would recommend all uni students participate if possible*”.

Christine, Female, 09:30

It is clear from Christine’s response that the experimental methodology had a strong impact on providing her with ‘food for thought.’ Furthermore, the findings suggest that this research could positively impact bystander intervention.

## 4. Discussion

This study developed an experimental methodology that moved away from measuring intent, which is not well linked to actual behaviour (McMahon et al., 2014), towards measuring actual bystander behaviour. Actors from the ATP team used invisible theatre to act out signs leading up to a sexual assault at a party constructed within the Immerse Studio. Using direct deception to ensure that natural intervening behaviours could be observed, participants were led to believe that they were there to evaluate the effectiveness of the Immerse Studio for hosting a party.

The first research question examined how participants responded to signs leading up to a sexual assault in a party environment. Reactions varied depending on the participant, situation, and their interpretation of events. Five participants did not notice any signs leading up to a sexual assault. Their focus was on the technology and socialising with other participants, as is typical in a party environment. Five participants did notice something suspicious; however, in line with past research (Burn, 2009), the perceived ambiguity of the situation inhibited intervention. The fear of misinterpreting deters bystanders from intervening (Labhardt et al., 2024).

Two females in the second session noticed something suspicious and intervened. They consistently monitored the situation between the victim and perpetrator. Their intervening behaviour ranged from indirect methods (e.g., non-verbal cues and photo-bombing a selfie) to direct intervention (i.e., following the victim and perpetrator out of the room) as the severity of the situation escalated. They described the situation to be clear, with no ambiguity. The two females who intervened did not know each other or the victim prior to participating. However, they came together to intervene as a team when witnessing the event. Based on the interviews, the positive peer support they had from each other gave them the strength needed to intervene, which also aligns with past research (Banyard et al., 2018).

Personal safety may also have been a factor for the two bystanders. In line with past research (Greitemeyer & Mügge, 2015), as the severity of the situation increased, the risk to personal safety increased for the two participants; as such, intervening together minimised this risk. Furthermore, research suggests that intervening behaviour can be prompted if bystanders share a similar social category group membership with each other and with the victim (Levine & Crowther, 2008; Levine et al., 2005). Perhaps, in this case, the shared group membership between the bystanders and the victim was their gender identity.

The findings regarding research question 1 are promising. However, as there were only two sessions, with intervention only occurring in one session (12:30), further testing is required. Developing the methodology to understand the circumstances in which people intervene individually compared to those who intervene with at least one other person could provide insight into the characteristics that comprise the bystander intervention phenomenon.

The second research question focused on how successful the methodology was in creating a believable party environment to test bystander behaviour via deception. During the interviews, prior to the disclosure of the deception, it was evident that participants were genuinely deceived about the project’s true nature. Evident in their authentic responses, participants focused solely on their experiences regarding the party environment. The music, people, activities, and food available in the Immerse Studio (commonly found at most parties) created a realistic party environment.

In developing this experimental methodology, it was possible to incorporate the extenuating aspects surrounding signs leading up to a sexual assault (Hughes, 1998). This addressed a limitation often associated with self-report data, where the situation’s complexity is not fully presented. The complexity of the environment created in the Immerse Studio provided a natural ‘distraction’, reducing direct attention towards the sexual assault in question, which is representative of what tends to occur at a real party. Overall, participants found the party environment to be a believable environment, even providing suggestions on how to develop it further while still under deception. The surrounding contexts were considered and are important when investigating bystander intervention. The deceptive design and the believable party environment created the indirect impact needed when measuring bystander intervention.

The third research question focused on how participants perceived the use of deception. Participants were not aware that they were being deceived and did not perceive the sexual assault cues to be abnormal in the environment, reducing the possibility of socially desirable responding. However, future research could consider an experimental design where some participants are fully aware of the study aim, while others are deceived to see if and how it affects bystander intervention. While deceptive research can cause mild distress in some participants (Bortolotti & Mameli, 2006), the effective utilisation of the funnelling debrief method mitigated the adverse effects of deception (Boynton et al., 2013). It allowed participants to be part of the conversation with the interviewer and the ATP actors, creating a safe space for all involved and providing a sense of being part of something.

During the funnelling debrief, most participants reported they were fine after the deception and the research aim was revealed. However, it is important to note that while most participants stated they were fine, this type of research does have its risks. For example, two participants spoke to either the social worker or the psychologist immediately following the interview, as they were upset about not intervening. Self-blame for not intervening is normal (Feldman & Albarracín, 2017), and reassurance was provided by the professionals onsite. Both participants stated that the reassurance and onsite support were beneficial. For this reason, support services must be available to ensure the well-being of all involved in deceptive research focusing on sexual assault. Furthermore, by adhering to the code of ethics and ensuring immediate support was available, participants left in a similar state to when they arrived, if not better (British Psychological Society, 2014). All participants were also emailed by the lead researcher three days after participating to do a wellness check and thank them for participating. No response came back from the two individuals who were upset about not intervening; there was no further follow-up. Furthermore, following participation, some participants reported feeling more confident and motivated to intervene in the future. Participants’ reports of a positive learning experience were an unintended yet welcome outcome from the research and suggest that this methodology may be used as an educational platform.

**Figure 5:**
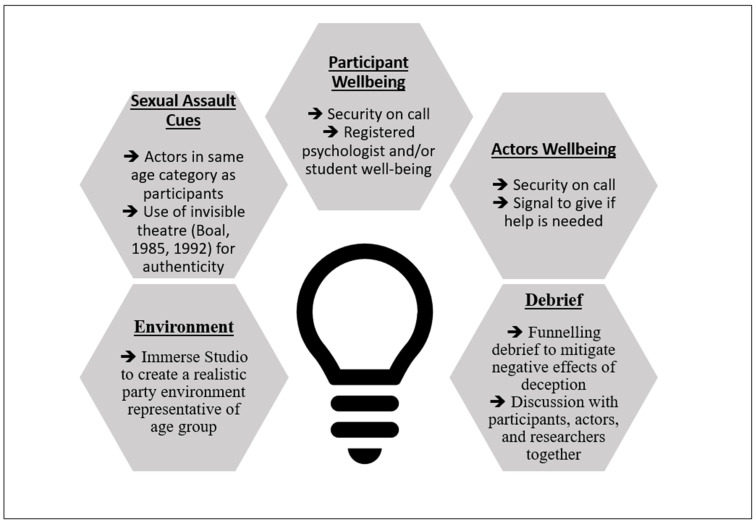
A visual guide on what to consider when designing an experimental methodology measuring actual bystander behaviour using deception.

Overall, using deception and implementing the funnelling debrief and onsite support appeared to be an effective way to examine actual bystander behaviour, within a lab-based setting, while mitigating the adverse effects associated with deception. However, it is important to develop and test this approach further. For a visual guide, please see Figure 5.

The paper’s findings need to be understood in the context of the research design. First, while every attempt was made at creating a realistic and naturalistic scenario as possible using deception and immersion, it was a lab-based study. Participants were aware they were taking part in a research project and that it was not an actual nightlife setting. As such, the observed intervention behaviours could be different to what occurs in a natural setting (e.g., night club). However, this study has made an important contribution in bridging the gap between self-report data to actual bystander behaviour. Future research could consider expanding on these findings and incorporating other methodologies, such as virtual reality, to continue developing this area of research. Second, time restrictions and a limited recruitment window resulted in a small sample size. Third, while participants reported being okay with the deception, some participants may have needed more time to process the information. Future research could include a formal follow-up with participants, further ensuring participant well-being. It could also provide insight into the long-term effects of deception and whether there are positive outcomes, as suggested by Schwartz and Gottlieb (1980). Fourth, participants were not screened in advance for any history of sexual violence. While the signs leading up to a sexual assault are not dissimilar to those encountered regularly within society, it could be triggering. In addition, the response to being deceived could be very different for those with lived experience. Involving survivors in the development of ethical protocols could be one way to address these concerns. Fifth, some participants knew each other and/or the actors. It is possible that this could have impacted their intervention behaviour. However, this was not explored or discussed in detail in the interviews. As such, future research should explore this further when utilising the experimental methodology. Sixth, a female victim and male narrative was used; however, future research could consider using a more inclusive, diverse narrative. Last, due to the novel approach, alcohol was not included due to ethical concerns. However, future research should consider the inclusion of alcohol, as it is a key element present at parties where a sexual assault could take place.

### Implications and Conclusions

These findings have important implications for extending the bystander literature and sexual assault prevention. First, the group discussions at the end of each session demonstrated that people tolerated the deception and were happy to participate in the research, even after the deception was revealed. Second, some participants reported that they felt their confidence and ability to intervene in the future increased, indicating that in vivo experiments may help build awareness and skills. Third, the participants found their involvement in the research study to constitute a positive learning experience. While not an intended outcome of the study, it demonstrated the positive impacts of educating students alongside building confidence. Expanding the experimental methodology to include additional variables such as alcohol could increase the complexity, similar to a real-life situation, and enhance this methodology for future research.

## Figures and Tables

**Table 2:** Frequencies for each observed bystander behaviour.

Code	0 (*n* = 5)	1 (*n* = 5)	2 (*n* = 1)	5 (*n* = 2)	Total (*n* = 13)
Gender					
Male Female	*n* = 3 *n* = 2	*n* = 1 *n* = 4	- *n* = 1	- *n* = 2	*n* = 4 *n* = 9

*Note*: 0 = See nothing, do nothing; 1 = see something, do nothing; 2 = see something, attention directed by another participant, no action taken; 5 = see something, direct action.

## Data Availability

The data are not publicly available due to privacy or ethical restrictions.
